# A Prospective Study of Grip Strength Trajectories and Incident Cardiovascular Disease

**DOI:** 10.3389/fcvm.2021.705831

**Published:** 2021-09-16

**Authors:** Weida Liu, Runzhen Chen, Chenxi Song, Chuangshi Wang, Ge Chen, Jun Hao, Yang Wang, Chenxi Yu

**Affiliations:** ^1^State Key Laboratory of Cardiovascular Disease, Fuwai Hospital, National Center for Cardiovascular Diseases, Peking Union Medical College & Chinese Academy of Medical Sciences, Beijing, China; ^2^Department of Cardiology, Fuwai Hospital, National Center for Cardiovascular Diseases, Peking Union Medical College & Chinese Academy of Medical Sciences, Beijing, China; ^3^Department of Joint Surgery, Shandong Provincial Hospital Affiliated to Shandong First Medical University, Jinan, China; ^4^Orthopaedic Research Laboratory, Medical Science and Technology Innovation Center, Shandong First Medical University & Shandong Academy of Medical Sciences, Jinan, China

**Keywords:** muscle function, trajectory, myocardial infarction, stroke, heart failure

## Abstract

**Background:** A single measurement of grip strength (GS) could predict the incidence of cardiovascular disease (CVD). However, the long-term pattern of GS and its association with incident CVD are rarely studied. We aimed to characterize the GS trajectory and determine its association with the incidence of CVD (myocardial infarction, angina, stroke, and heart failure).

**Methods:** This study included 5,300 individuals without CVD from a British community-based cohort in 2012 (the baseline). GS was repeatedly measured in 2004, 2008, and 2012. Long-term GS patterns were identified by the group-based trajectory model. Cox proportional hazard models were used to examine the associations between GS trajectories and incident CVD. We identified three GS trajectories separately for men and women based on the 2012 GS measurement and change patterns during 2004–2012.

**Results:** After a median follow-up of 6.1 years (during 2012–2019), 392 participants developed major CVD, including 114 myocardial infarction, 119 angina, 169 stroke, and 44 heart failure. Compared with the high stable group, participants with low stable GS was associated with a higher incidence of CVD incidence [hazards ratio (HR): 2.17; 95% confidence interval (CI): 1.52–3.09; *P* <0.001], myocardial infarction (HR: 2.01; 95% CI: 1.05–3.83; *P* = 0.035), stroke (HR: 1.96; 95% CI: 1.11–3.46; *P* = 0.020), and heart failure (HR: 6.91; 95% CI: 2.01–23.79; *P* = 0.002) in the fully adjusted models.

**Conclusions:** The low GS trajectory pattern was associated with a higher risk of CVD. Continuous monitoring of GS values could help identify people at risk of CVD.

## Introduction

Muscle dysfunction reduces the quality of life and increases healthcare budgets. Many studies suggest that muscle dysfunction predisposes to the development of diabetes (1), hypertension (2), and cardiovascular disease (CVD) (3, 4). Therefore, monitoring muscle function is essential to improve quality of life. However, the exact measurement of muscle content requires consuming and costly techniques, such as magnetic resonance imaging or dual-energy X-ray absorptiometry techniques (5–7); meanwhile, muscle content might not fully reflect muscle function (8). Muscle strength, as measured by grip strength (GS), has been shown to be a simple, inexpensive indicator of CVD in population studies (9).

CVDs are known as the leading causes of non-communicable disease-related deaths and one of the most serious health problems worldwide (10), which caused nearly one-third of all deaths worldwide (11). Universally, CVD can refer to a kind of disease that involves the blood vessels or heart (12). This disease consists of myocardial infarction (MI), stroke, heart failure, and many other vascular and cardiac problems (12). In the past few decades, the adverse impacts of CVD have been improved. However, CVD is still responsible for a huge reduction in quality of life and imposes remarkable expenditures on health systems in different countries (13).

Previous cohort (9, 14, 15) and Mendelian randomization (16) studies have consistently shown that muscle strength was a risk factor for CVD incidence. Data from the Prospective Urban Rural Epidemiology (PURE) study of nearly 140,000 participants from 613 communities in 17 countries showed that lower GS is associated with a higher risk of CVD incidence (9). Another study (17) conducted in the UK investigated the changes in GS to be associated with mortality, but GS has only been measured twice in 4 years, and that study did not investigate whether changes in GS are associated with CVD. So far, there is a lack of research on the GS trajectory and its relationship with CVD. Besides, few studies have simultaneously reported the association between GS and MI, angina, stroke, and heart failure. This might be partly due to the different goals of diverse studies, but it could also be due to data-driven emphasis about which outcome to report.

In view of these knowledge gaps, this study aimed to identify GS trajectories over 8 years among 5,300 English participants and to explore the relationship between long-term GS patterns and the subsequent CVD risk. We further investigated the association of GS trajectories with the components of the CVD events, including MI, angina, stroke, and heart failure. We hypothesized that (a) people have different GS patterns and (b) people with different GS patterns would be at different levels of risk for the incidence of CVD.

## Methods

### Study Design and Participants

Data were obtained from the English Longitudinal Study of Ageing (ELSA). ELSA is a nationally representative, biannual, ongoing, longitudinal cohort study based on community-dwelling adults living in England (18). The detailed descriptions of the study design, sampling procedure, and data collection were published previously (18, 19).

**Figure 1:**
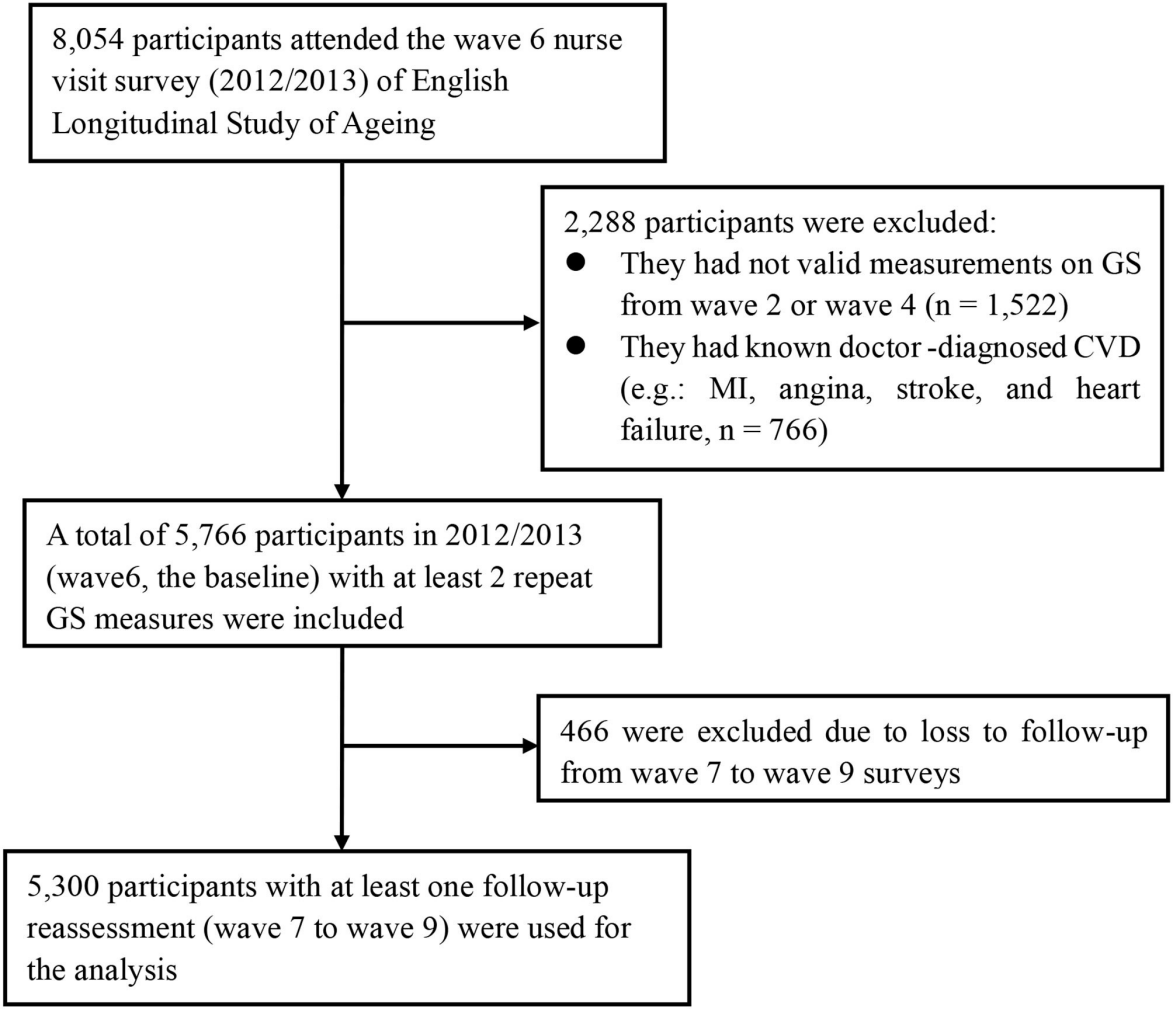
Flowchart of participant selection for the present study population. CVD, cardiovascular disease; MI, myocardial infarction; GS, grip strength.

The current study used data from wave 2 (2004/2005) to wave 9 (2018/2019). Every 4 years from 2004 (wave 2) through 2012 (wave 6), GS was measured by trained nurses. Participants were excluded if they did not have valid GS measurements between 2004 and 2008 (wave 2 or wave 4, *n* = 1,522) or if they already had a doctor-diagnosed CVD (i.e., MI, angina, stroke, and heart failure, *n* = 766) at the baseline (i.e., 2012). In addition, we excluded participants who were lost to follow-up from wave 7 to wave 9 (*n* = 466). Finally, a total of 5,300 participants were included in 2012 (the baseline), with at least two repeat GS measures and at least one follow-up reassessment (during 2012–2019) for the current analysis of CVD risk. The participant selection flowchart is shown in Figure 1.

All waves of the ELSA received ethical approval from the London Multicenter Research Ethics Committee (MREC/01/2/91), and all participants provided informed consents.

### Objective Measurement of Grip Strength

The Smedley handheld dynamometer (Stoelting Co., Wood Dale, IL, USA) was used to measure GS (kg) three times on each hand. The participant was asked to squeeze the device for a couple of seconds and to rest at least 30 s between each measurement. The overall GS value was computed as the average of maximum GS of the non-dominant and dominant hands.

### Assessment of Covariates

Covariates shown by previous studies to be associated with both CVD and GS were selected for our study, including age, sex, body mass index (BMI), education, current smoking, physical activity, sleep quality, depression, hypertension, and diabetes. BMI (kg/m^2^) was calculated based on body weight and height measured in light clothing without shoes. Education level was classified as (1) equivalent or less than the general certificate of education (GCE) O-level, (2) equivalent or GCE O-level, and (3) higher than GCE A-level or equivalent. Smoking status was categorized as non-smokers (never smoked or ex-smokers) and current smokers. Physical activity was divided into two levels based on whether once or more times moderate or vigorous exercises a week. Sleep quality was grouped as good (score ≤ 2) or bad (score >2) using the Jenkins sleep scale. The level of depression was evaluated by the Centre for Epidemiological Studies Depression scale (CES-D). A score ≥4 denoted a higher level of depression. The BPs were measured three times using an electronic sphygmomanometer (Omron HEM-907, Omron Corporation, Kyoto, Japan) after resting for more than 5 min, and the average value of the three consecutive readings was used in the analyses. Hypertension was diagnosed as individuals with a mean SBP ≥140 mmHg and/or mean DBP ≥90 mmHg, or individuals who reported hypertension or received antihypertensive treatment. Diabetes was defined as individuals who reported diabetes or use insulin/antidiabetic medications.

### Outcomes

The primary outcome of the current study was the incidence of CVD, defined as the composite outcome of self-reported physician-diagnosed MI, angina, stroke, and heart failure. The secondary outcomes were the components of the primary composite outcome. The incidence of CVD was defined as the report of physician-diagnosed from wave 7 to wave 9. The date of diagnosis of the CVD event is recorded between the date of the last interview and the date of the interview reporting the CVD event. The researchers of ELSA had collected further information of incident CVD events from the medical records to confirm the diagnosis.

### Statistical Analysis

To distinguish the heterogeneity of CVD by investigating the trajectory of the GS pattern before the CVD event, we applied the group-based trajectory model (GBTM) analysis (20–22) to categorize participants with similar GS change patterns through the TRAJ procedure in SAS. The GBTM could classify the participants with a similar GS pattern into a group automatically and fit the GS measurements in each group according to a generalized linear mixed model. Considering GS values change with time, the GBTM used in our study is useful because it simulates the group-specific trajectories of GS. In our study, GS patterns from 2004 to 2012 were modeled by the latent class method. As GS varies largely according to gender and age, GS was modeled separately for men and women as well as adjusting for age. Up to five trajectory groups were set in advance. We fitted the model from one group trajectory to five group trajectories, and visit wave (years) was used as the time scale. The censored normal model was used appropriately for continuous outcomes. To identify the model with optimal functional forms of distinct GS trajectories, starting from the highest polynomial, the cubic, quadratic, and linear terms were considered and assessed according to the significance level. Model fit was compared using the Bayesian information criterion (BIC) and Akaike's information criterion (AIC) value, with the smallest negative number indicating the best fit model. We described GS from 2004 to 2012 by mean [standard deviation (SD)].

Multivariable Cox proportional hazard models were used to analyze the association between GS patterns from 2004 to 2012 and the risk of CVD. The proportionality of hazards was checked by the Schoenfeld residuals method. Results of Cox models were presented as hazard ratios [HRs; 95% confidence intervals (CIs) and *P*-values]. All analyses are presented as the two models with adjustment of confounding factors in 2012. These confounders shown by previous studies to be associated with both CVD and GS were selected for our analyses. More specifically, the minimally adjusted model was adjusted for age and sex. The fully adjusted model was further adjusted for education, BMI, systolic blood pressure, smoking, physical activity, depression, and quality of sleep.

For the effects of sex or age, likelihood ratio tests were performed to explore statistical interactions between GS patterns and age ( ≤ 65 vs. >65) and GS patterns and sex of primary outcome, respectively, by comparison of the −2 logarithmic likelihood chi-square between the nested model with and without a product term. Stratified models by sex and age ( ≤ 65 vs. >65) were utilized to explore the associations between trajectories and CVD risks. We conducted all analyses with SAS version 9.4 (Cary, NC, USA).

## Results

**Figure 2:**
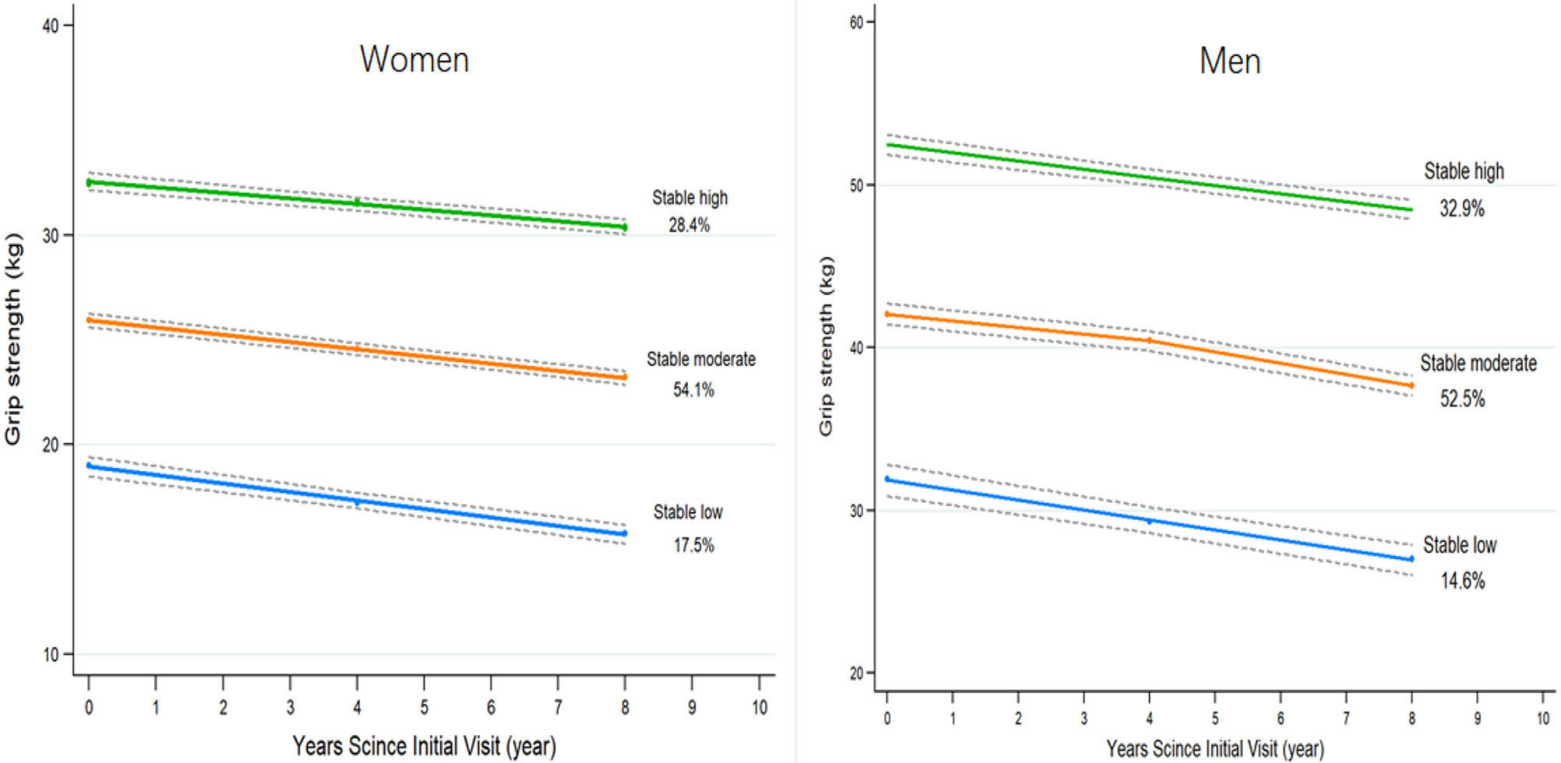
The trajectories of grip strength by sex predicted with 95% confidence intervals. Three groups are indicated by stable high (green), stable moderate (orange), and stable low (blue). Percentages represent the proportion of the cohort with the corresponding group number as the highest predicted posterior probability.

The model with three trajectories according to the change patterns of GS from 2004 to 2012 was identified as the fit by comparing the BIC and the proportion of the participants within each trajectory group (Figure 2). For women, 507 participants were classified as low stable GS (15.8–19.0 kg), 1,656 as moderate stable GS (23.2–26.0 kg), and 820 as high stable GS (30.3–32.4 kg). For men, 325 participants were classified as low stable GS (27.0–31.9 kg), 1,241 as moderate stable GS (37.7–42.1 kg), and 751 as high stable GS (48.4–52.3 kg) (Supplementary Tables 1–3).

Table 1 shows the baseline characteristics of participants in each GS trajectory group. Compared with the low stable GS group, participants with high and moderate stable patterns of GS tend to be younger, have lower systolic BP and higher BMI and diastolic BP, and have a higher prevalence of levels of education above the GCE A-level or equivalent, moderate–vigorous activity, and good sleep quality. The proportion of participants who reported current use of tobacco, non-diabetes, and non-hypertension was the highest in the stable high GS group, followed by the moderate stable GS and low stable GS groups.

**Table 1:** Baseline characteristics of the participants based on trajectories of grip strength.

**Variables**	**Total (*N* = 5,300)**	**Stable low grip strength (*N* = 832)**	**Stable moderate grip strength (*N* = 2,897)**	**Stable high grip strength (*N* = 1,571)**	***P***
Age (years)	68.0 ± 8.3	76.3 ± 9.6	68.2 ± 7.2	63.2 ± 5.4	<0.0001
Women	2,983 (56.3)	507 (60.9)	1,656 (57.2)	820 (52.2)	<0.0001
Education					<0.0001
Less than GCE O-level	2,039 (38.6)	437 (52.9)	1,121 (38.8)	481 (30.8)	
GCE O-level	1,490 (28.2)	205 (24.8)	829 (28.7)	456 (29.2)	
Higher than GCE A-level	1,750 (33.2)	184 (22.3)	939 (32.5)	627 (40.1)	
Body mass index (kg/m^2^)	27.2 ± 7.2	25.6 ± 8.8	27.3 ± 7.0	28.1 ± 6.4	<0.0001
Systolic blood pressure (mmHg)	132.7 ± 17.1	134.1 ± 18.7	132.9 ± 17.2	131.6 ± 16.2	0.0090
Diastolic blood pressure (mmHg)	73.8 ± 10.3	70.0 ± 10.8	73.8 ± 10.2	76.0 ± 9.7	<0.0001
Current smoking	516 (9.7)	62 (7.5)	284 (9.8)	170 (10.8)	0.0293
Moderate-vigorous activity	3,470 (65.5)	370 (44.5)	1,960 (67.7)	1,140 (72.6)	<0.0001
Good sleep quality	4,204 (79.4)	617 (74.2)	2,301 (79.6)	1,286 (81.9)	<0.0001
Depressive symptoms	344 (6.5)	46 (5.5)	182 (6.3)	116 (7.4)	0.1712
Hypertension	1,733 (32.7)	355 (42.7)	963 (33.2)	415 (26.4)	<0.0001
Diabetes	420 (7.9)	100 (12.0)	231 (8.0)	89 (5.7)	<0.0001

*Data are expressed as number (%) or mean (standard deviation)*.

During a median follow-up of 6.1 years (interquartile range: 5.8–6.3 years), we recorded 392 CVD events, with 114 MI, 119 angina, 169 stroke, and 44 heart failure. In comparing high vs. low, low stable GS was associated with a higher risk of CVD incidence during 2012–2019. Table 2 shows the HR for CVD incidence (HR: 2.29; 95% CI: 1.64–3.20; *P* <0.001) was greater in individuals with low stable GS compared with the high stable GS in age/sex-adjusted models. The association remained significant (HR: 2.17; 95% CI: 1.52–3.09; *P* <0.001) after adjustment for further confounding factors. Compared with the stable high group, participants in the stable low GS group had a double significantly higher risk of CVD incidence.

**Table 2:** Cox regression analyses for CVD incidence per trajectory of grip strength (2004–2012).

	**Trajectories of grip strength**			***P* for interaction**
	**Stable high**	**Stable moderate**	**Stable low**	
**Total population**				
No. of events (%)	81/1,571 (5.2)	194/2,897 (6.7)	117/832 (14.1)	
**HR (95% CI)**				
Age/sex adjusted	1 (ref)	1.17 (0.89–1.53)	2.29 (1.64–3.20)	
Fully adjusted^a^	1 (ref)	1.13 (0.85–1.51)	2.17 (1.52–3.09)	
Stratified by sex				0.462
**Women**				
No. of events (%)	34/820 (4.2)	81/1,656 (4.9)	67/507 (13.2)	
HR (95% CI)				
Age/sex adjusted	1 (ref)	1.04 (0.69–1.56)	2.23 (1.37–3.63)	
Fully adjusted	1 (ref)	0.99 (0.64–1.52)	2.05 (1.22–3.43)	
**Men**				
No. of events (%)	47/751 (6.3)	113/1,241 (9.1)	50/325 (15.4)	
**HR (95% CI)**				
Age/sex adjusted	1 (ref)	1.30 (0.91–1.86)	2.33 (1.47–3.69)	
Fully adjusted	1 (ref)	1.26 (0.86–1.85)	2.23 (1.37–3.63)	
Stratified by age				0.572
**≤65 years**				
No. of events (%)	56/1,114 (5.0)	60/1,171 (5.1)	10/112 (8.9)	
**HR (95% CI)**				
Age/sex adjusted	1 (ref)	1.10 (0.76–1.59)	1.95 (0.99–3.83)	
Fully adjusted	1 (ref)	1.08 (0.73–1.59)	1.90 (0.95–3.81)	
**>65 years**				
No. of events (%)	25/457 (5.5)	134/1,726 (7.8)	107/720 (14.9)	
**HR (95% CI)**				
Age/sex adjusted	1 (ref)	1.56 (1.02–2.39)	3.66 (2.37–5.67)	
Fully adjusted	1 (ref)	1.49 (0.96–2.34)	3.47 (2.18–5.52)	

*Data are expressed as n (%) or hazard ratio (95% CI). For this analysis, the group with stable high trajectories of grip strength was used as the reference (ref) and compared with all the other groups*.

*CVD, cardiovascular disease; NA, not available; HR, hazard ratio; CI, confidence interval*.

a *Fully adjusted = adjusted for age, sex, education level, body mass index, systolic blood pressure, smoking, physical activity, depression, and sleep quality*.

Results stratified by age and sex are shown in Table 2. Associations between GS trajectories and CVD risk were generally consistent in the subgroup analysis, as no significant interactions were observed in terms of age (*P*-interaction = 0.572) and gender (*P*-interaction = 0.462). Besides, for individuals with older age (>65 years), low and moderate GS was associated with a more substantial increment of CVD risk (Table 2), as compared with younger participants (age ≤ 65 years). Furthermore, low GS was associated with significantly higher CVD risk in individuals aged >65 years, but lost its statistical significance (*P* = 0.572) in individuals aged ≤ 65 years.

Table 3 examines the associations of GS trajectories with the components of the CVD events. In the age/sex-adjusted model, low stable GS was positively associated with MI, angina, stroke, and heart failure incidence, respectively. The results remained similar after adjusting for additional covariates, except for the incidence of angina (fully adjusted HR: 1.73; 95% CI: 0.93, 3.19; *P* = 0.083). Compared with the high stable GS group, individuals with stable low GS had a higher risk of MI (fully adjusted HR: 2.01; 95% CI: 1.05, 3.83; *P* = 0.035), stroke (fully adjusted HR: 1.96; 95% CI: 1.11, 3.46; *P* = 0.020), and heart failure (fully adjusted HR: 6.91; 95% CI: 2.01, 23.79; *P* = 0.002), respectively.

**Table 3:** Cox regression analyses for the components of the CVD events per trajectory of grip strength (2004–2012).

	**Trajectories of grip strength**		
	**Stable high**	**Stable moderate**	**Stable low**
**Myocardial infarction**			
No. of events (%)	28/1,571 (1.8)	56/2,897 (1.9)	30/832 (3.6)
**HR (95% CI)**			
Age/sex adjusted	1 (ref)	1.07 (0.67–1.72)	2.01 (1.14–3.83)
Fully adjusted^a^	1 (ref)	1.10 (0.67–1.80)	2.01 (1.05–3.83)
**Angina**			
No. of events (%)	32/1,571 (2.0)	56/2,897 (1.9)	31/832 (3.7)
**HR (95% CI)**			
Age/sex adjusted	1 (ref)	0.96 (0.61–1.50)	1.94 (1.08–3.47)
Fully adjusted	1 (ref)	0.80 (0.49–1.29)	1.73 (0.93–3.19)
**Stroke**			
No. of events (%)	30/1,571 (1.9)	85/2,897 (2.9)	54/832 (6.5)
**HR (95% CI)**			
Age/sex adjusted	1 (ref)	1.24 (0.80–1.90)	2.12 (1.25–3.60)
Fully adjusted	1 (ref)	1.32 (0.83–2.10)	1.96 (1.11–3.46)
**Heart failure**			
No. of events (%)	4/1,571 (0.3)	24/2,897 (0.8)	14/832 (1.7)
**HR (95% CI)**			
Age/sex adjusted	1 (ref)	3.18 (1.08–9.34)	3.90 (0.97–8.65)
Fully adjusted	1 (ref)	6.58 (1.95–22.20)	6.91 (2.01–23.79)

*Data are expressed as n (%) or hazard ratio (95% CI). For this analysis, the group with stable high trajectories of grip strength was used as the reference (ref) and compared with all the other groups*.

*CVD, cardiovascular disease; NA, not available; HR, hazard ratio; CI, confidence interval*.

a *Fully adjusted = adjusted for age, sex, education level, body mass index, systolic blood pressure, smoking, physical activity, depression, and sleep quality*.

In addition, we have also added relevant data on the association between baseline grip strength and CVD risk in Supplementary Table 4.

## Discussion

Muscles, as the main sites for protein storage and glucose processing, play an important role in maintaining the health of people (23, 24). GS was chosen as a biomarker of muscular fitness given its sensitivity to physiological change and its use as a valid marker of muscle function (25). In this English cohort study from 2004 to 2019, we explored the different GS patterns (2004–2012) before the diagnosis of CVD. By using GBTM, we identified three GS groups. The incidence of CVD was highest in the low stable GS group (14.1%), followed by the moderate stable (6.7%) and high stable (5.2%) groups. As far as we know, this is the first prospective cohort study regarding the impacts of longitudinal GS patterns on the incidence of CVD.

The main finding of our study was that stable low GS was strongly associated with a wide range of CVD outcomes. Several studies (9, 14, 26–28) have documented the associations between GS and risk of CVD, where GS was only measured once at baseline. Our results showed that people with the low GS trajectory pattern had a double higher risk of CVD compared with those in the stable high GS group in the fully adjusted model. The associations observed were consistent between the sexes. Interpretations could be multiple regarding how muscle function decline could lead to the incidence of CVD. Musculoskeletal dysfunction may lead to a reduction in muscular contraction-inducing factors (also known as myokines) with anti-inflammatory effects (29), which increases the risk of developing CVD and relevant complications. Besides, endothelial dysfunction, autonomic imbalance, and arterial stiffness might mediate the relationship between CVD and muscle strength (28). Our data suggest that long-term GS patterns might be used to screen patients during physical examination, which has remarkable implications for primary healthcare practice. The general practitioners could stratify individuals given the values of GS change and prescribe physical activities to enhance the general muscular fitness of people with stable low GS, which may serve as an essential part in the management and prevention of CVD. Therefore, there is a demand for increasing recognition that the long-term GS value can be used as a useful clinical biomarker in a health monitoring system.

In the analysis by age subgroup, the association between GS patterns and CVD incidence was more obvious among participants aged >65 years compared with those aged ≤ 65 years. However, the interaction between age and GS trajectories for CVD was not statistically significant. Previously, few studies have investigated whether the associations between CVD and GS are consistent across age groups. A systematic review (30) including 53,476 individuals from 14 studies showed that the relationship between GS and mortality appeared to be weaker in people under 60 years, but the interaction with age was not formally verified due to the low number of available studies. Our results showed that in the elderly, the risk of CVD in the stable low GS population is 3.5 times that of the stable high GS population. Our findings are important for CVD prevention in the elderly, especially for the elderly with stable low GS. A future study is warranted to investigate whether improved muscle strength could directly lower the risk of CVD.

Furthermore, we examined the association of GS trajectories with the components of the CVD events. The predicted 6-year risk of MI and stroke increased substantially in the group of stable low GS. In the PURE study (9), a large longitudinal population study, baseline GS was more predictive of adverse health outcomes than SBP. It has also been shown that every 5 kg decline in GS was associated with a 7% increase in the risk of MI and a 9% higher increase in stroke. In addition, our research also suggested that stable low GS people were associated with a higher risk of heart failure. However, the accuracy of the estimation of HR value could be less accurate due to the rare incidence of heart failure and the wide confidence interval. Collectively, these data indicate that long-term GS testing should be used as a first-line screening for identifying people at high risk of a wide range of CVD events.

To our knowledge, the current study was the first to investigate the association between GS trajectory and the risk of CVD among a nationally representative English population. The prospective design and high follow-up rate minimized the possibility of recall bias and follow-up loss, allowing the capture of a considerable number of CVD events. Our study design involved repeated GS measurements, which enabled us to create the trajectory of GS change and obtain the GS performance of participants in the years before CVD diagnosis. Because we excluded participants with diagnosed CVD at baseline and used longitudinal GS changes instead of one measurement of GS, reverse causality bias is unlikely in our study.

Despite the strengths mentioned above, our study still has several limitations. First, the use of self-reported physician-diagnosed CVD could cause misclassifications of CVD events and might influence the results to a lesser extent. However, most CVD cases identified in our study were confirmed by ELSA researchers based on medical records. Second, the number of GS measurements is relatively less and the study may not acquire sufficient power to depict the full trajectories of GS. Defining the trajectory of GS changes based on three measurements within 8 years does not seem optimal. However, the systematic measurement of longitudinal GS data is already precious. Third, the use of data from the responders might have influenced our results, as non-responders might have a higher incidence of CVD and an accelerated rate of GS change compared with responders. Finally, although we have attempted to minimize potential confounding in the analyses, the findings might still be affected by the residual confounders from unknown and unmeasured factors (such as inflammation markers, lipid levels, and diet).

## Conclusion

In summary, the long-term GS pattern was associated with the altered risk of CVD. People with stable low GS had twice the risk of developing CVD as those with stable high GS. When stratifying by age, the association was more pronounced in the older population. These results suggest that continuous GS values could be utilized to identifying people at risk of CVD, especially in the elderly. Furthermore, our research indicates that high stable muscle function may reduce the risk of CVD, which provides the potential to prevent and treat the condition.

## Data Availability Statement

The original contributions generated for the study are included in the article/Supplementary Material, further inquiries can be directed to the corresponding author/s.

## Author Contributions

WL carried out the concepts, design, data analysis, and manuscript. RC, CY, and CW reviewed and polished the drafts. JH and CS provided assistance for data acquisition. The final manuscript was approved by all authors.

## Funding

The current study was funded by the National Key Research and Development Plan Project Tasks, Project No: 2020YFC1107902.

## Conflict of Interest

The authors declare that the research was conducted in the absence of any commercial or financial relationships that could be construed as a potential conflict of interest.

## Publisher's Note

All claims expressed in this article are solely those of the authors and do not necessarily represent those of their affiliated organizations, or those of the publisher, the editors and the reviewers. Any product that may be evaluated in this article, or claim that may be made by its manufacturer, is not guaranteed or endorsed by the publisher.
