# Supplementary material for: A Prospective Study of Grip Strength Trajectories and Incident Cardiovascular Disease
Source: Front Cardiovasc Med. 2021 Sep 16;8:705831. doi: 10.3389/fcvm.2021.705831 (PMC8481887; doi:10.3389/fcvm.2021.705831)
Supplement: Supplementary file 1 [file Data_Sheet_1.docx]

| Visit wave (Year) | Trajectories of grip strength (kg) | | |
| --- | --- | --- | --- |
|  | Low stable | Moderate stable | High stable |
| Women |  |  |  |
| Wave 2 (2004/2005) | 19.0 (18.5 – 19.4) | 26.0 (25.6 – 26.3) | 32.4 (32.2 – 33.0) |
| Wave 4 (2008/2009) | 17.2 (17.0 – 17.7) | 24.5 (24.3 – 24.8) | 31.6 (31.2 – 31.8) |
| Wave 6 (2012/2013) | 15.8 (15.3 – 16.2) | 23.2 (22.9 – 23.5) | 30.3 (30.0 – 30.8) |
| Men |  |  |  |
| Wave 2 (2004/2005) | 31.9 (30.9 - 32.8) | 42.1 (41.4 - 42.7) | 52.3 (51.8 - 53.1) |
| Wave 4 (2008/2009) | 29.3 (28.6 - 30.2) | 40.4 (39.8 - 41.0) | 50.7 (50.0 - 51.0) |
| Wave 6 (2012/2013) | 27.0 (26.0 - 27.9) | 37.7 (37.1 - 38.3) | 48.4 (47.9 – 49.1) |

**APPENDIX**

**Supplementary Table 1 The range of GS trajectories from 2004 to 2012**

Grip strength was expressed as mean (95% CI) except where otherwise stated.

**Supplementary Table 2 Fit statistics for grip strength group trajectories by sex**

| Fit statistic | Number of groups | | | | | | |
| --- | --- | --- | --- | --- | --- | --- | --- |
|  | 1 | 2 | 3 | | 4 | | 5 |
| Women | | | | | | | |
| BIC* | -24753.66 | -23491.22 | -22984.14 | -22806.91 | | -22737.28 | |
| AIC* | -24744.66 | -23470.22 | -22951.14 | -22761.90 | | -22680.27 | |
| Group percent | Group1, 100% | Group1, 43.5% | Group1,17.5% | Group1, 9.1% | | Group1,5.7% | |
|  |  | Group2, 56.5% | Group2,54.1% | Group2,34.5% | | Group2,22.5% | |
|  |  |  | Group3,28.4% | Group3,41.5% | | Group3,41.1% | |
|  |  |  |  | Group4,14.9% | | Group4,26.3% | |
|  |  |  |  |  | | Group5,4.4% | |
| Men | | | | | | | |
| BIC* | -21427.24 | -20394.70 | -20036.74 | -19833.16 | | -19785.14 | |
| AIC* | -21418.62 | -20374.58 | -20002.25 | -19790.05 | | -19730.54 | |
| Group percent | Group1, 100% | Group1, 46.3% | Group1,14.6% | Group1, 8.7% | | Group1,8.4% | |
|  |  | Group2, 53.7% | Group2,52.5% | Group2,39.6% | | Group2,0.4% | |
|  |  |  | Group3,32.9% | Group3,41.5% | | Group3,39.4% | |
|  |  |  |  | Group4,10.2% | | Group4,41.4% | |
|  |  |  |  |  | | Group5,10.4% | |

AIC Akaike’s information criterion, BIC Bayesian information criteria,

*A lower absolute value suggests a better model fit

**Supplementary Table 3 The final three-group trajectory model of grip strength by sex**

| Trajectory group | Parameter | Maximum likelihood estimates | | | |
| --- | --- | --- | --- | --- | --- |
|  |  | Est. | SE | *Z* value | *P* value |
| Women | | | | | |
| Group1: low stable grip strength (n=507, 17.5%) | Intercept | 18.95 | 0.24 | 79.78 | < 0.001 |
|  | Linear | -0.40 | 0.03 | -11.79 | < 0.001 |
| Group2: moderate stable grip strength (n=1,656, 54.1%) | Intercept | 25.94 | 0.16 | 158.28 | < 0.001 |
|  | Linear | -0.34 | 0.02 | -16.91 | < 0.001 |
| Group3: high stable grip strength (n=820, 28.4%) | Intercept | 32.57 | 0.21 | 153.86 | < 0.001 |
|  | Linear | -0.27 | 0.03 | -9.14 | < 0.001 |
| Men | | | | | |
| Group1: low stable grip strength (n=325, 14.6%) | Intercept | 31.85 | 0.49 | 64.60 | < 0.001 |
|  | Linear | -0.61 | 0.06 | -9.47 | < 0.001 |
| Group2: moderate stable grip strength (n=1,241, 52.5%) | Intercept | 42.07 | 0.33 | 128.06 | < 0.001 |
|  | Linear | -0.27 | 0.12 | -2.27 | 0.024 |
|  | Quadratic | -0.03 | 0.01 | -2.40 | 0.016 |
| Group3: high stable grip strength (n=751, 32.9%) | Intercept | 52.46 | 0.32 | 166.07 | < 0.001 |
|  | Linear | -0.50 | 0.06 | -10.97 | < 0.001 |

Est. parameter estimate, SE standard error of parameter estimate

**Supplementary Table 4 Cox regression analyses for the CVD events by grip strength quartile in baseline**

|  | | **Grip strength group** ^a^ | | | | | |
| --- | --- | --- | --- | --- | --- | --- | --- |
|  | | **High** | | **Moderate** | | **Low** | |
| **CVD** | |  | |  | |  | |
| **No. of events (%)** | | 68/1286 (5.3) | | 195/2802 (7.0) | | 129/1212 (10.6) | |
| **HR (95%CI)** | |  | |  | |  | |
| **Age/sex adjusted** | | 1 (ref) | | 1.17 (0.88-1.55) | | 1.48 (1.07-2.05) | |
| ^b^**Fully adjusted** | | 1 (ref) | | 1.20 (0.89-1.63) | | 1.49 (1.06-2.11) | |
| **Myocardial infarction** |  | |  | |  | |  |
| **No. of events (%)** | | 29/1286 (2.3) | | 49/2802 (1.8) | | 36/1212 (3.0) | |
| **HR (95%CI)** | |  | |  | |  | |
| **Age/sex adjusted** | | 1 (ref) | | 0.74 (0.46-1.19) | | 1.11 (0.64-1.92) | |
| **Fully adjusted** | | 1 (ref) | | 0.75 (0.46-1.24) | | 1.09 (0.61-1.94) | |
| **Angina** |  | |  | |  | |  |
| **No. of events (%)** | | 26/1286 (2.0) | | 57/2802 (2.0) | | 36/1212 (3.0) | |
| **HR (95%CI)** | |  | |  | |  | |
| **Age/sex adjusted** | | 1 (ref) | | 0.99 (0.62-1.60) | | 1.36 (0.78-2.36) | |
| **Fully adjusted** | | 1 (ref) | | 0.93 (0.56-1.54) | | 1.34 (0.75-2.41) | |
| **Stroke** | |  | |  | |  | |
| **No. of events (%)** | | 21/1286 (1.6) | | 91/2802 (3.3) | | 57/1212 (4.7) | |
| **HR (95%CI)** | |  | |  | |  | |
| **Age/sex adjusted** | | 1 (ref) | | 1.61 (0.99-2.62) | | 1.72 (0.99-3.00) | |
| **Fully adjusted** | | 1 (ref) | | 1.89 (1.10-3.26) | | 1.84 (1.00-3.36) | |
| **Heart failure** | |  | |  | |  | |
| **No. of events (%)** | | 2/1286 (0.2) | | 24/2802 (0.9) | | 16/1212 (1.3) | |
| **HR (95%CI)** | |  | |  | |  | |
| **Age/sex adjusted** | | 1 (ref) | | 5.19 (1.21-22.17) | | 7.00 (1.54-31.89) | |
| **Fully adjusted** | | 1 (ref) | | 4.97 (1.15-21.39) | | 6.39 (1.37-29.70) | |

CVD: cardiovascular disease. NA: not available. HR: hazard ratio. CI: confidence interval.

^a^ Because GS varies greatly between sexes and age groups, we categorized gender-and age-specific GS into low (<25^th^ percentile), moderate (25^th^-75^th^ percentile), and high (>75^th^ percentile) strength subgroups using their respective 25^th^ and 75^th^ percentile values as a cutoff point.

^b^ Fully adjusted=adjusted for age, sex, education, body mass index, Systolic blood pressure, smoking, physical activity, depression, and sleep quality.

Data are expressed as n (%) or hazard ratio (95% CI) except where otherwise stated. The crude event rates are shown. For this analysis, the group with Stable high Trajectories of grip strength was used as the reference (ref) group and compared with all other groups.
